# The effect of deep brain stimulation on motor and cognitive symptoms of Parkinson's disease: A literature review

**DOI:** 10.1590/S1980-57642015DN91000005

**Published:** 2015

**Authors:** Flavia Amaral Machado, Caroline Tozzi Reppold

**Affiliations:** 1 Physiotherapist, Master’s Student of Master’s Program of Rehabilitation Sciences – Universidade Federal de Ciências da Saúde de Porto Alegre (UFCSPA), Brazil.; 2 PhD, Psychologist, Neuropsychological Assessment Unit, Master’s Program of Rehabilitation Sciences and Health Sciences. Universidade Federal de Ciências da Saúde de Porto Alegre (UFCSPA), Brazil.

**Keywords:** Parkinson's disease, deep brain stimulation (DBS), cognition, motor symptoms

## Abstract

Deep brain stimulator (DBS) implant surgery is considered a breakthrough in the treatment of Parkinson's disease, especially in cases where motor symptoms cannot be controlled through conventional drug treatment. Its benefits have been studied extensively in the literature, particularly in relation to motor symptoms. However, the disease's cognitive aspects have been studied to a lesser extent.

**Objective:**

This systematic review aims to assess the effects of DBS surgery on motor and cognitive symptoms in patients with Parkinson's disease.

**Methods:**

The search strategy included MEDLINE, LILACs, SCIELO and the Cochrane Library. Randomized clinical trials with DBS surgical intervention and Parkinson's disease were included. Of the 178 studies identified, 19 met the eligibility criteria. These studies were descriptively analyzed as regards to their results.

**Results:**

Control of motor symptoms, as assessed by the UPDRS Part III scale, was found in all of the studies, pointing to great interest in this outcome and demonstrating an advantage of DBS over conventional drug treatment. Regarding cognitive aspects, heterogeneity in the choice of subjects studied and the use of different assessment tools for each was evident, hampering comparisons and leading to inconclusive results.

**Conclusion:**

This review provides a broad overview of the effects of DBS on Parkinson's disease symptoms. However, it is suggested that future studies be conducted to establish a gold-standard protocol for neuropsychological assessment, thereby enabling data comparison and more consistent conclusions.

## Abstract (pt)

A cirurgia de implante de estimulador encefálico profundo (DBS) é considerada um avanço em termos de tratamento para doença de Parkinson, especialmente em casos nos quais o controle dos sintomas motores não é possível por meio de tratamento farmacológico convencional. Os benefícios são bastante estudados na literatura, especialmente no que se refere aos sintomas motores. Contudo, os aspectos cognitivos também envolvidos nessa doença vêm sendo estudados em menor escala.

**Objetivo:**

O objetivo dessa revisão sistemática é verificar os efeitos da cirurgia DBS nos sintomas motores e cognitivos de pacientes com a Doença de Parkinson.

**Métodos:**

A estratégia de busca inclui MEDLINE, LILACS, SCIELO, Biblioteca Cochrane. Ensaios clínicos randomizados com a intervenção cirúrgica DBS e Doença de Parkinson foram incluídos. Dos 178 estudos identificados, 19 cumpriram os critérios de elegibilidade, os quais foram analisados de forma descritiva quanto aos seus resultados.

**Resultados:**

O controle dos sintomas motores avaliados através da escala UPDRS, seção III, foi encontrado em todos os estudos, evidenciando o grande interesse por esse desfecho e demonstrando uma vantagem do DBS em comparação com o tratamento convencional medicamentoso. Em relação aos aspectos cognitivos, existe uma heterogeneidade na escolha dos domínios estudados e a utilização de diferentes instrumentos de avaliação para cada um deles, dificultando a comparação e tornando os resultados inconclusivos.

**Conclusão:**

Essa revisão fornece um panorama amplo dos efeitos do DBS nos sintomas da Doença de Parkinson, no entanto sugere-se que futuros estudos estabeleçam um protocolo de avaliação neuropsicológica padrão-ouro para permitir confrontação dos dados e conclusões mais consistentes.

## INTRODUCTION

Parkinson's disease (PD), first described by the English doctor James Parkinson in 1817, is considered a neurodegenerative disease, highly prevalent in the elderly population worldwide, whose incidence tends to increase with the population aging observed in recent years. Its main features are associated with motor symptoms of bradykinesia, resting tremor, postural instability and muscle rigidity. However, non-motor symptoms also affect the individual and are responsible for limitations and disabilities that impair the quality of life in these individuals.^1,2^

The neuropathology of PD involves the degeneration of dopaminergic neurons located in the brain's substantia nigra. It is a process in which neurons are lost in the region called the pars compacta and results from the accumulation of proteins (mainly alpha-synuclein) in this region. The loss of dopaminergic terminals leads to a decrease in dopamine transporter (DAT) density. When symptoms are present, this reduction in DAT density can reach 90% of normal levels.^3-5^

The main characteristic motor symptoms of PD are resting tremor, slow movement, muscular rigidity and postural instability. Secondary symptoms include freezing and problems with handwriting. Contrary to James Parkinson's initial description, PD presents a range of associated non-motor symptoms. These include changes in relation to smell, cognitive/behavioral aspects, sleep disorders and others, all of which greatly interfere with patients' general health and have a major impact on their quality of life.^6-8^ For this reason, these symptoms have been the focus of recent studies.

PD treatment is based on dopaminergic replacement, with levodopa being the most commonly used drug.^9^ A precursor of dopamine, levodopa's emergence in the late 1960s was considered a landmark in the treatment of patients with PD. However, despite its positive effects, its extended use can lead to problems related to motor fluctuations and dyskinesia.^3^ These results vary according to duration of levodopa use and disease progression. Therefore, it is generally preferred to delay initial levodopa treatment in order to postpone the onset of these related effects. Cases also exist in which patients do not respond to drug treatment or in which they develop intolerance to the drug, making treatment a challenge for medicine.^10^

In this context, deep brain stimulation (DBS) represents one of the most important innovations for PD treatment. Many studies have shown that this type of surgical treatment can significantly improve the motor condition of individuals with fluctuations and dyskinesia.^11,12^ There is abundant information on the results of short-term (up to one year) implants and a considerable number of medium-term studies (from one to five years) on the disease's motor aspects. To a lesser extent, studies have assessed post-operative cognitive aspects.^13^ These studies have shown that DBS leads to a marked and sustained improvement in dopaminergically responsive motor symptoms, with a reduction in dyskinesia severity and duration. There is a drastic reduction in the need for dopaminergic medication after implant,^14^ leading to a decrease in dyskinesia.

The main objective of this article was to present a systematic review of the literature, especially of the randomized clinical trials on the effect of deep DBS implant surgery on PD motor and cognitive symptoms. More specifically, the aim was to identify whether the implant surgery improved motor symptoms and resulted in changes (improvement/deterioration) in any specific area of cognitive function.

## METHODS

**Search strategy.** The following electronic databases were searched: MEDLINE (accessed through PubMed), LILACS and the Cochrane Library. We used the research terms 'deep brain stimulation', 'Parkinson disease' and 'randomized controlled trial'. The search period was not limited and included all articles published up to September 2013. The exclusion criteria were: duplicate studies; lack of data or incomplete data on results obtained or sample results; and outcomes other than those described in the eligibility criteria.

**Study eligibility.** We included studies in English, Portuguese and Spanish that were designed as randomized clinical trials with DBS surgery as the main intervention in individuals with PD, having outcomes of control of motor symptoms and cognitive aspects (verbal fluency, memory, attention and executive functions). Other outcomes of interest were quality of life and mood (behavioral aspects).

**Study selection and data extraction.** The titles and abstracts of all the articles identified by the search strategy were evaluated. All abstracts that did not provide sufficient information on inclusion and exclusion criteria had their associated full texts read in order to evaluate them. In this research stage, the reviewers independently assessed the entire articles and made their selections according to the eligibility criteria, collecting data on the interventions, outcomes, instruments used for evaluation and methodological characteristics. Disagreements among the reviewers were resolved by consensus.

**Methodological quality.** Evaluation of the studies' quality was based on the GRADE approach, as recommended by the Cochrane Handbook for Systematic Reviews of Interventions.^16^ This was done independently by two reviewers and included analysis of the following: generation of appropriate randomization; allocation concealment; blind patients and investigators; blinding of result evaluators; use of intention-to-treat analysis; and descriptions of losses and exclusions. Studies lacking a clear description of their randomization process and those concealing their allocation lists (including terms such as "randomization by telephone", "based on the Web" and "central") were considered not to have satisfied these criteria. Intention-to-treat analysis was considered after confirming that the participants who were randomized and analyzed were identical, except for those who were lost during follow-up or who withdrew their consent to participate in the study. Three other factors were analyzed to shed light on the studies' quality:

[1] study design limitations;
[2] consistency of results; and
[3] objectivity.

All of the aspects considered were assessed as "adequate" or "inadequate".

**Analyses.** The analysis was descriptive for outcomes found and type of instrument used to assess motor and cognitive aspects. Furthermore, methodological characteristics and main evidence according to the central aim of the research were reported.

## RESULTS

**Figure 1:**
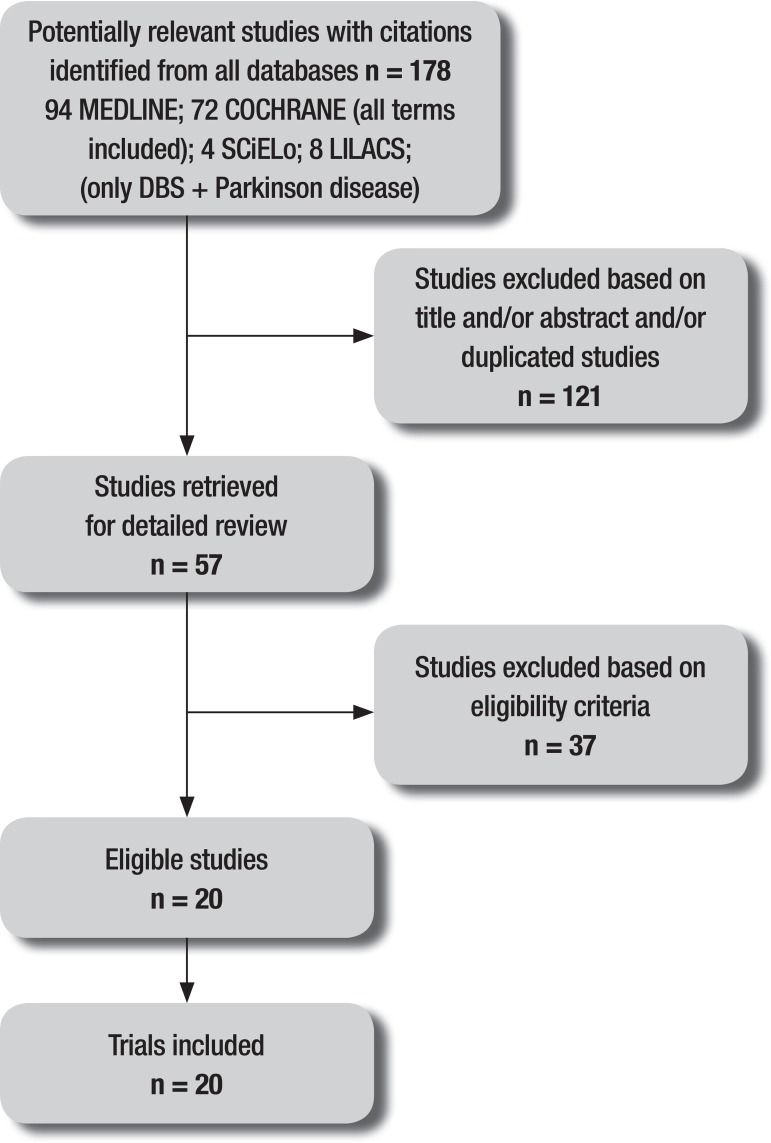
Flow diagram of studies included in the review.

The initial search identified 166 studies using all of the search terms. An additional 12 studies were found using only two search terms ('Parkinson's disease' and 'deep brain stimulation') in two databases, giving a total of 178 studies. Of these, 57 were selected to have their full texts read and 19 satisfied the present review's eligibility criteria. The complete flow chart of studies included in this review is given in Figure 1. The included studies involved a total of 2,180 patients.

Study quality was classified as good in half the studies, according to the assessment based on the GRADE approach as recommended by the Cochrane Handbook for Systematic Reviews of Interventions.^16^ With regard to the impact factor of the journals in which the studies were published, a range of 1.161 to 50.807 was found. All of these are indexed in the ISI Web of Knowledge database.

As for the proposed intervention, 42% of the studies had controlled clinical trials performed with a sample divided into two groups: a surgical group that underwent surgery to implant a deep brain stimulator in the subthalamic nuclei (DBS-STN) and another group that received best medical treatment, considering the drug treatment used.^11,17-19,22-25^ Follow-up ranged from six to 24 months.

Cognitive aspects were assessed using various types of instruments, covering specific cognitive areas (attention, memory, executive functions and verbal fluency) or cognitive functions in general. The Mattis Dementia Rating Scale (MDRS)^26^ was the most commonly used instrument (used by researchers in 50% of the studies to assess cognitive functions overall), followed by the Semantic Category Verbal Fluency Test and the Phonemic Category Verbal Fluency Test (37.5%).^27^ The Digit Span Test for memory and the Stroop Test^28^ for attention were used in two studies (25%). The analysis of the data concerning the effects of surgery on cognitive symptoms suggested no adverse effects except on verbal fluency (VF), which showed a greater decline after DBS (semantic VF declined 4.5 points and phonemic VF declined 3.06 points) compared with drug treatment.^17^

Witt et al.^18^ studied the relationship between the trajectory of the electrodes and the neuropsychological assessment results and found increased risk of a decline in global cognition and working memory performance in electrode trajectories involving caudate nuclei. Furthermore, the DBS group exhibited a decline in verbal fluency in 75% of the patients and a significant difference when compared with the group that received drug treatment (DBS= -6.1 points and Med=0.3 points).

**Table 1:** Study results.

Study, year	Participants N (male/female)	Treatment applied =N	Mean age (years)	Duration of disease (years)	Follow-up (months)	Number of assessments (period)
Burchiel et al., 1999	10 (7m/3f)	DBS GPi=4 DBS Stn=6	56	12.1	0-12m	5 (preop, 10 days, 3,6,12 months)
Daniels et al., 2011	121 (m/f=NI)	DBS Stn=61 Med=60	59.7	13	0-6m	2 (baseline ,6 months)
Deuschl et al., 2006	156 (100m/56f)	DBS Stn=78 Med=78	60.6	13.4	0-6m	2 (baseline ,6 months)
Deuschl et al., 2013	251 (m/f=NI)	DBS Stn+Med=123 Med=123	52.6	7.5	0-24m	3 (5,12,24 months)
Follet et al., 2010	299 (249m/50f)	DBS GPi=152 DBS Stn=147	61	11.3	0-24m	5 (3,6,12,18,24 months)
Gill et al., 2011	30 (27m/3f)	Med=15 DBS Stn+Med=15	60	2.2	0-24 m	5 (baseline, 6,12,18, 24 months)
Merello et al., 2008	16 (9m/7f)	DBSStn+Sub=5 DBS Stn Bil=6 Sub Bil=5			0-12m	3 (1,6,12 months)
Odekerken et al., 2013	128 (88m/38f)	DBS Stn:63 DBS GPi:65	60	11	0-12m	4 (1,3,6,12 months)
Okun et al., 2009	45 (m/f)	DBS GPi=23 DBS Stn=22	60	13	0-7m	2 (preop and 7 months)
Rocchi et al., 2012	66 (m/f=NI)	HC=28 PDC=9 DBS Stn=15 DBS GPi=14	61	12	0-6 m	3 (preop, baseline and 6 months)
Schubpach et al., 2007	20 (12m/8f)	DBS Stn=10 Med=10	48	6.8	0-18m	4 (preop, 6, 12, 18 months)
Smeding et al., 2005	34 (11m/23f)	Palli=14 DBS Stn=20	61	11.5	0-12m	3 (base, 6, 12 months)
Weaver et al., 2009	255 (206m/49f)	DBS GPi=61 DBS Stn=60 med=134	62	11	0-6m	2 (base, 6 months)
Weaver et al., 2012	156 (131m/25f)	DBS GPi=89 DBS Stn=70	60.5	11	0-36m	6 (3,6,12,18,24,36 months)
Weiss et al., 2013	12 (9m/3f)	DBS Stn=6 DBS Stn+SNi=6	65	>4.5	0-6 weeks	3 (baseline, 3 and 6 weeks)
Williams et al., 2010	366 (256m/110f)	DBS Stn=183 Med=183	59	11.3	0-12m	2 (baseline, 12 months)
Witt et al., 2008	123 (77m/46f)	DBS Stn=60 Med=63	59.5	13.9	0-6 m	2 (baseline, 6 months)
Witt et al., 2013	62 (28m/34f)	DBS Stn=31 Med=31	59.3	>5	0-6 m	2 (baseline, 6 months)
Zahodne et al., 2009	42 (30m/12f)	DBS GPi=22 DBS Stn=20	61.3	13	0-6m	2 (baseline, 6 months)

N: number of subjects; m: male; f: female; NI: not informed; DBS GPi: Deep Brain Stimulation of the globus pallidus internal; DBS Stn: Deep Brain Stimulation of subthalamic nucleus; DBS SNi: Deep Brain Stimulation of substantia nigra; Palli: Pallidotomy; Sub: Subthalamotomy; Bil: Bilateral; Med: Best Medical Treatment; HC: Healthy Control; PDC: Parkinson’s Disease Control; Preop: Presurgery; Baseline.

Quality of life outcomes were also assessed in 100% of the studies, using the Parkinson's Disease Questionnaire (PDQ-39)^29^ in all assessments. The quality of life results were better for those patients who underwent DBS. In the Daniels et al study,^22^ the main objective was to evaluate the quality of life of patients who underwent DBS-STN surgery, unlike other studies that predominantly aimed to assess the control of motor symptoms. Most of the scores on the quality of life questionnaires improved considerably after DBS-STN and the analysis showed a threshold of 10.9 points for the PDQ-39, indicating a change in quality of life. In the study by Schubpack et al.,^24^ 24% of those who underwent the surgery showed better results on the activities of daily living, stigma and body discomfort subareas of the rating scale, reporting this improvement after 18 months of follow-up. There was no difference in outcome for the group that underwent drug treatment only.

The results for motor symptom control were assessed using the Unified Parkinson's Disease Rating Scale (UPDRS),^30^ section III, which covers the motor aspects related to the main PD symptoms, such as bradykinesia, gait, rigidity, gait freezing, language and facial expression, among others. On this scale, a higher score indicates greater motor impairment in the individual. In the study by Gill et al.,^23^ the group that underwent surgical stimulation had a reduction of four points compared to baseline scores after two years. A similar result was found in the study by William et al.,^25^ which found a reduction of 6.6 points after one year of surgery, while the group undergoing drug treatment had an increase of 1.6 points after the same period. Confirming this trend, Deuschl et al.,^11^ showed results of 71% in favor of surgical treatment and only 21% for drug treatment. Even within this context, it can be stated that all of the studies that compared DBS-STN with drug treatment showed better motor symptom control for those who underwent DBS-STN surgery.

Regarding the surgical target, comparisons between the brain locations in DBS surgery [implantation in the subthalamic nuclei (STN) vs. the internal globus pallidus (GPi)] and their effects on motor and cognitive symptoms were found in 31.5% of the studies, whose follow-up periods ranged from six to 36 months. Increased control of motor symptoms proved similar between the two implant groups, as the studies found no significant differences and the results showed an improvement ranging from 26% to 40% for STN and 28% to 40% for GPi.^20,31,32^ UPDRS section III was used as the assessment tool in all studies.

The quality of life of patients was measured in four (66.6%) out of the six trials that compared the surgical implant locations. PDQ-39 was the tool most used (75%) but results were conflicting since Weaver et al.^33^ and Follet et al.^21^ found no difference or insignificant differences, whereas in other studies quality of life improved more for the GPi group than for the STN group (38% vs. 14% respectively; p=0.03), suggesting a greater improvement for the DBS-GPi group.^20,31^

Regarding cognitive symptoms, similarly to that described above, to compare the surgical and drug treatment groups, the areas of verbal fluency, attention, memory and overall cognitive function were assessed. The evaluations were performed using the following tools: the Mattis Dementia Rating Scale (MDRS), the Wechsler Adult Intelligence Scale (WAIS III), the Stanford-Binet Intelligence Scale for overall cognitive function in two (33.3%) of the studies, the Wisconsin Card Sorting Test (WCST) for executive functions in one study, and the Hopkins Verbal Learning Test (HVLT) for memory in two studies. The only cognitive area that showed significant decline was verbal fluency, which was evaluated in three studies using semantic and phonemic verbal fluency tests. Okun et al.^31^ presented evidence that, between the pre- and post-surgery results, there was a greater worsening in the phonemic verbal fluency test score for the DBS-STN group (STN -5.8±10.0vs. GPi -3.1±7.6, p<0.05). Zahodne et al.,^20^ who also found a greater worsening in the DBS-STN group (phonemic verbal fluency: pre-surgery 37.9 (9.9) vs. post-surgery 32.5 (10.03) p<0.001), pointed out that their study did not provide conclusive evidence that the GPi is a safer target. The results for the other areas assessed did not indicate significant changes and were considered consistent with PD's process of progression. However, the study by Weaver et al.^33^ concluded that cognitive decline does not differ according to the surgical target. Although significant differences were detected on the Hopkins and Mattis dementia test results, these were attributed to differences in unadjusted results and considered confounding factors, as shown in the total Mattis Scale score data (on which a higher score indicates better performance): GPi (n.89) vs. STN (70)/Baseline: 137.8±5.1vs. 137.1±4.8; GPi (n.86) vs. STN (66) / 36-months: 135.2±8.7x 130.9±13.2.

In another prior study, Weaver et al.^12^ performed a comparison of three groups: the two surgical groups mentioned previously plus a drug treatment group. All groups had short-term follow-ups of six months. A battery of neuropsychological assessments composed of the following tools was applied: the Mattis Dementia Rating Scale (MDRS), Wechsler Adult Intelligence Scale (WAIS-III), semantic and phonemic verbal fluency tests, Wisconsin Card Sorting Test (WCST), Boston Naming Test, Brief Visuospatial Memory Test, Hopkins Verbal Learning Test (HVLT) and the Stroop test for attention. The neurocognitive tests revealed small decreases of between 1.0 and 3.5 points in some areas of information processing for patients who received DBS compared to those who received medical treatment.

Compared with the drug treatment group, the DBS group showed significant improvement on seven of the eight subscales measuring quality of life according to the PDQ-39 results. The results in elderly patients were greatest for mobility (-12.3), activities of daily living (-14.0) and stigma (-12.5), considering the difference between the baseline and six-month assessments and bearing in mind that a higher score indicates worse quality of life for the individual. The group treated with medication showed little change from baseline, except in the stigma subarea (-4.2).

The motor results showed a significant improvement in the DBS group compared with the medical treatment group, as 71% of the DBS patients and only 32% of the drug therapy patients had better motor function scores, according to the UPDRS III. These results contradict those found by Rocchi et al.,^34^ who compared the two surgical targets (STN and GPi) and two control groups (one with PD patients and one composed of healthy individuals) and found similar scores for the surgical and control groups, with PD: 45.5±16 and 22.9±8.9at baseline and 45.9±12.^8^ and 24.2±9.6after six months (off and on medication, respectively).

Three other studies eligible for this review reported controlled trials with different surgical approaches between groups. One of these, by Weis et al.,^35^ entailed a comparison using DBS-STN patients as the control group and DBS-STN plus substantia nigra (SN) patients as the combined therapy group, showing improvement in both surgical groups (from baseline to three weeks later) for motor symptom control, according to the UP-DRS III. There was no significant difference between the two groups: STN+SNi: 13 (6.47); STN: 14.25 (5.75); effect=0.83 (3.86); 95% confidence interval (CI) -1.-3.82; (P=0.470). However, determining the incidence of neurocognitive interference in the stimulation of the SN combined with the STN requires cohort studies with greater numbers of participants and longer follow-up periods to obtain more robust conclusions, while the trial in question lasted for only three weeks and had only 12 participants.

The second study, by Merello et al.,^36^ compared three surgical groups: bilateral DBS-STN, bilateral subthalamotomy and unilateral DBS-STN with contralateral subthalamotomy (total n=16). These also showed improvement in motor symptoms, as assessed by the UPDRS III. However, sample size analysis showed that 16 individuals in each group would be necessary to demonstrate significant differences. Neuropsychological assessment tools were used to evaluate six areas: attention, memory, verbal fluency, orientation, language and visuospatial skills. However, this was a pilot study and its results cannot be considered sufficiently consistent to infer any kind of conclusion.

Finally, Smeding et al.^37^ conducted a study with 12 months of follow-up, comparing a group that underwent DBS-STN and a group that underwent pallidotomy, evaluating them using a battery of neurocognitive assessment tools including the following tests: the Dementia Rating Scale (DRS), semantic and verbal fluency tests, the Dutch Adult Reading Test (DART), Controlled Oral Word Association Test (COWAT), Paced Auditory Serial Addition Task (PASAT), auditory verbal learning test (AVLT), Groningen Intelligence Test, Stroop Test, Odd Man Out Test (OMO), Trail Making Test and the Boston Naming Test (BNT).

The results indicated no significant difference between the DBS-STN and pallidotomy groups on the main neuropsychological assessments, although the DBS-STN group committed more errors than the pallidotomy group on two executive tests (the Stroop and Trail Making tests) six months after surgery. The effect size was large (Cohen Stroop Test d=0.94; Cohen Trail Making Test Part B d=0.80), suggesting impairment in executive functions. After 12 months, there was a trend toward decreased verbal fluency performance. Although the two groups did not differ significantly in degree of decline in verbal fluency, the DBS-STN group exhibited a greater decrease (2.6 points). The results of this study are in line with those of Witt et al.,^17,18^ which found a higher error rate on the Stroop Test for the bilateral DBS-STN group after six months. The authors concluded that bilateral STN stimulation has slightly more negative effects on executive function than unilateral pallidotomy.

## DISCUSSION

The improvement in motor conditions in PD patients who underwent deep brain stimulator implant surgery is well established in the literature. Through this review, 18 articles were found that assessed motor symptoms, on/off time and control of symptoms such as dyskinesias and motor fluctuations. These studies used different approaches and different comparison groups, and their evaluation periods ranged from those that included pre-surgical patients to those evaluating patients 36 months after intervention. The common factor in these studies was the positive result of surgery compared to that of drug treatment in controlling motor symptoms. However, the studies do not present evidence that either the STN or GPi is better or worse as a surgical target.

Even within this context, the improvement in quality of life after DBS is derived from motor symptom control factors, and the length of time a patient stayed in the 'off' condition proved to be the most important predictor of surgical results. The most commonly used tool was the PDQ-39 questionnaire, which has the advantages of being an assessment tool for a specific validated disease, having 39 questions divided into eight areas, covering mobility, activities of daily living, emotional well-being, social support, body discomfort, stigma, cognition and communication.

However, significant gaps remain in understanding of the effects of DBS on cognitive functions. This literature review dealing with DBS and cognitive aspects evaluated the articles, concluding that the greatest difficulty in establishing the impact of DBS surgery on cognitive aspects was due to the diversity of tools used for each area, hampering comparison of studies. Taking the example of motor and quality of life assessments (in which most studies used the UPDRS III and PDQ-39, respectively), the unification of tools for cognitive functions in PD could be an advantageous and effective way to comparing results for this population. This review identified only verbal fluency, as measured by the semantic and phonemic verbal fluency tests,^12,17,18,20,23,31,33,36,37^ as an area exhibiting decline after DBS, a finding more evident in cases where the surgery targeted the subthalamic nucleus (STN). The moderate decline in semantic and phonemic verbal fluency after DBS was described by Parsons et al.,^38^ who reviewed 28 cohort studies investigating the effect of DBS on the cognitive aspects of 612 patients. The explanation for this result remains unclear. However, the decline in executive functions after DBS-STN could be due to an effect on basal ganglia circuits. Moreover, in the Witt et al. study,^18^ which assessed electrode trajectory, the authors suggested that the caudate nucleus could be spared by this trajectory, preferring a more anterior-lateral path that would be cognitively safer.

Another important aspect of this review is the fact that it did not establish a single type of intervention as an eligibility criterion, leading to the inclusion of a larger number of studies, which provided a much broader scope in terms of surgical effects on patients' motor and cognitive symptoms, affording a study covering six different types of controlled clinical trials encompassing all the studies available in the databases. However, this became an obstacle for the reviewers when preparing the results and comparing them across studies. It was therefore decided to present the results by type of intervention. Furthermore, the variation in the number of patients in the study samples was considered large, ranging from 10^32^ to 366,^25^ demonstrating the heterogeneity of the studies found.

Finally, this review covered the most important studies conducted to date^11,19,21,25,31^ and presented some important considerations about the effects of DBS surgery on PD patients. However, some limitations should be noted, such as the diversity of tools used in the studies for assessing the cognitive areas, which led to less consistent results. Another limiting factor was the small number of studies (1 or 2) for some intervention types, demonstrating that much more research is needed to establish the safety and effectiveness of this procedure on cognitive and behavioral functions. Further controlled clinical trials should be conducted with groups eligible for DBS surgery employing different targets, as well as with control groups in use of drug treatment.

## References

[1] Rowland LP (2007). Merrit- Tratado de Neurologia.

[2] Levy A, Ferreira J (2003). Doença de Parkinson - Manual Prático.

[3] Teixeira Jr AL, Cardoso F (2004). Tratamento inicial da doença de Parkinson. Rev Neurociênc.

[4] Vasconcellos LFR, Novis SAP, Moreira DM, Rosso ALZ, Leite ACC (2009). Neuroimaging in Parkinsonism: a study with magnetic resonance and
spectroscopy as tools in the differential diagnosis. Arq Neuropsiquiatr.

[5] Weng YH, Yen TC, Chen MC (2004). Sensitivy and specific of 99m Tc- TRODAT-1 SPECT imagining in
differentiating patients with idiopathic Parkinson's disease from healthy
subjects. J Nucl Med.

[6] Peternella N, Magalhães F, Marcon SS (2012). Quality of life of a person with Parkinson's disease and the
relationship between the time of evolution and the severity of the
disease. Rev. Latino-Am. Enfermagem.

[7] Silva JAM, Dibai Filho AV, Faganello FR (2011). Measurement of quality of life for individuals with Parkinson's
disease through the questionnaire PDQ-39. Fisioter mov.

[8] Silva TBL, Yassuda MS, Guimarães VV, Florindo AA (2011). Verbal fluency and sociodemographic variables in the aging
process: an epidemiological study. Psicol Reflex Crit.

[9] Melo LM, Barbosa ER, Caramelli P (2007). Cognitive impairment and dementia in Parkinson's disease:
clinical characteristics and treatment. Rev Psiq Clín.

[10] Tosta ED, Rieder CRM, Borges V (2010). Doença de Parkinson: Recomendações.

[11] Deuschl G, Schade-Brittinger C, Krack P (2006). A randomized trial of deep-brain stimulation for Parkinson's
disease. N Engl J Med.

[12] Weaver FM, Follett K, Stern M (2009). Bilateral deep brain stimulation versus best medical therapy for
patients with advanced Parkinson disease: a randomized clinical
trial. JAMA.

[13] Limousin P, Krack P, Pollak P (1998). Electrical stimulation of the subthalamic nucleus in advanced
Parkinson's disease. N Engl J Med.

[14] Romito LM, Contarino MF, Vanacore N, Bentivoglio AR, Scerrati M, Albanese A (2009). Replacement of dopaminergic medication with subthalamic nucleus
stimulation in Parkinson's disease: long-term observation. Mov Disord.

[15] Fasano A, Romito LM, Daniele A (2010). Motor and cognitive outcome in patients with Parkinson's disease
8 years after subthalamic implants. Brain.

[16] Higgins J, Green S (2008). Cochrane Handbook for Systematic Reviews of Interventions.

[17] Witt K, Daniels C, Reiff J (2008). Neuropsychological and psychiatric changes after deep brain
stimulation for Parkinson's disease: a randomised, multicentre
study. Lancet Neurol.

[18] Witt K, Granert O, Daniels C (2013). Relation of lead trajectory and electrode position to
neuropsychological outcomes of subthalamic neurostimulation in Parkinson's
disease: results from a randomized trial. Brain.

[19] Deuschl G, Schüpbach M, Knudsen K (2013). Stimulation of the subthalamic nucleus at an earlier disease
stage of Parkinson's disease: Concept and standards of the
EARLYSTIM-study. Parkinsonism Relat Disord.

[20] Zahodne LB, Okun M S, Foote KD (2009). Greater improvement in quality of life following unilateral deep
brain stimulation surgery in the globus pallidus as compared to the
subthalamic nucleus. J Neurol.

[21] Follett KA, Weaver FM, Stern M (2010). Pallidal versus subthalamic deep-brain stimulation for
Parkinson's disease. N Engl J Med.

[22] Daniels C, Krack P, Volkman J (2011). Is Improvement in the Quality of Life After Subthalamic Nucleus
Stimulation in Parkinson's Disease Predictable. Mov Disord.

[23] Gill CE, Allen LA, Konrad PE (2011). Deep Brain Stimulation for Early Stage Parkinson's Disease: An
Illustrative Case. Neuromodulation.

[24] Schupbach WM, Maltete D, Houeto JL (2007). Neurosurgery at an earlier stage of Parkinson disease A
randomized, controlled trial. Neurology.

[25] Williams A, Gill S, Varma T (2010). Deep brain stimulation plus best medical therapy versus best
medical therapy alone for advanced Parkinson's disease (PD SURG trial): a
randomised, open-label trial. Lancet Neurol.

[26] Mattis S, Odessa FL (1988). Dementia rating scale.

[27] Spreen O, Strauss E (1991). A compendium of neuropsychological tests.

[28] Stroop JR (1935). Studies of interference in serial verbal
reactions. J Exp Psychol.

[29] Jenkinson C, Heffernan C, Doll H, Fitzpatrick R (2006). The Parkinson's disease questionnaire (PDQ-39): evidence for a
method of imputing missing data. Age Ageing.

[30] Fahn S, Elton RL, Fahn S, Marsden CD, Calne D, Goldstein M (1987). Unified Parkinson's disease rating scale. Recent developments in Parkinson's disease.

[31] Okun MS, Fernandez HH, Wu SS (2009). Cognition and mood in Parkinson's disease in subthalamic nucleus
versus globus pallidus interna deep brain stimulation: the COMPARE
trial. Ann Neurol.

[32] Burchiel K, Anderson V, Favre J, Hammerstad F (1999). Comparison of pallidal and subthalamic nucleus deep brain
stimulation for advanced Parkinson's disease: results of a randomized,
blinded pilot study. Neurosurgery.

[33] Weaver FM, Follett KA, Stern MB (2012). Randomized trial for deep brain stimulation for Parkinson's
disease: 36 month outcomes. Neurology.

[34] Rocchi L, Carlson-Kuhta P, Chiari L, Burchiel KJ, Hogarth P, Horak FB (2012). Effects of deep brain stimulation in the subthalamic nucleus or
globus pallidus internus on step initiation in Parkinson disease: laboratory
investigation. J Neurosurg.

[35] Weiss D, Walach M, Meisner C (2013). Nigral stimulation for resistant axial motor impairment in
Parkinson's disease? A randomized controlled trial. Brain.

[36] Merello M, Tenca E, Perez Lloret S (2008). Prospective randomized 1-year follow-up comparison of bilateral
subthalamotomy versus bilateral subthalamic stimulation and the combination
of both in Parkinson's disease patients: a pilot study. Br J Neurosurg.

[37] Smeding HM, Esselink BS, Schmand B (2005). Unilateral pallidotomy versus bilateral subthalamic nucleus
stimulation in PD A comparison of neuropsychological effects. J Neurol.

[38] Parsons TD, Rogers SA, Braaten AJ, Woods SP, Troster AI (2006). Cognitive sequelae of subthalamic nucleus deep brain stimulation
in Parkinson's disease: a meta-analysis. Lancet Neurol.

